# LRP6 promotes invasion and metastasis of colorectal cancer through cytoskeleton dynamics

**DOI:** 10.18632/oncotarget.22759

**Published:** 2017-11-30

**Authors:** Qian Yao, Yu An, Wei Hou, Ya-Nan Cao, Meng-Fei Yao, Ning-Ning Ma, Lin Hou, Hong Zhang, Hai-Jing Liu, Bo Zhang

**Affiliations:** ^1^ Department of Pathology, School of Basic Medical Sciences, Peking University Health Science Center, Beijing 100191, China; ^2^ Department of Pathology, Beijing Tongren Hospital, Capital Medical University, Beijing 100730, China

**Keywords:** colorectal cancer, Wnt signaling, LRP6, metastasis, cytoskeleton

## Abstract

Low density lipoprotein (LDL) receptor-related protein-6 (LRP6) is an important co-receptor of Wnt pathway, which plays a predominant role in development and progression of colorectal cancer. Recently, dysregulation of LRP6 has proved to be involved in the progression of cancers, but its biological role and clinical significance in colorectal cancer remain unclear. In present study, we revealed that phosphorylation of LRP6 was aberrantly upregulated in colorectal carcinoma correlating with TNM or Dukes staging and worse prognosis. In addition, phosphorylated LRP6 was positively correlated with nuclear accumulation of β-catenin. Overexpression or activation of LRP6 could activate Wnt signaling and promote tumor cell migration *in vitro*. The activation of LRP6 could induce microtubule dynamics and actin remodeling, probably through regulation of microtubule-associated protein 1B (MAP1B), microtubule actin cross-linking factor 1 (MACF1) and Rho GTPase--RhoA and Rac1. The investigation suggests that LRP6 may be a potential prognostic marker and therapeutic target in the progression of colorectal cancers.

## INTRODUCTION

Colorectal cancer is the third most common cancer and fourth leading cause of cancer-related deaths worldwide [1, 2] and there is potential for distant metastasis following surgical excision of the primary lesion [3]. Despite the introduction of new treatments, the 5-year survival rate for metastatic colorectal cancer remains below 10% [4]. Hence, the underlying mechanism of metastasis, predictive biomarkers and therapeutic targets for colorectal cancer are under extensive exploration. Loss of function mutation in *APC* (adenomatous polyposis coli) tumor suppressor gene has been detected in about 85% of sporadic colorectal cancers, which leads to activation of Wnt signaling pathway [5]. In fact, activation of Wnt signaling has been clarified to be involved in initiation and progression of colorectal cancers in past decades. Especially, nuclear translocation of β-catenin, a hallmark of Wnt signaling activation, has proved to be a critical role in many aspects of cellular biological activities, such as growth, differentiation, EMT, as well as cytoskeleton remodeling [6, 7]. Nevertheless, Wnt signaling could be further enhanced by its secreted ligands even in APC-mutated situation, suggesting that that hyperactivation of Wnt pathway may be contributed by other alterations in colorectal cancers [8-11].

Low-density lipoprotein (LDL) receptor-related protein-6 (LRP6) is one of the co-receptors of Wnt pathway, which form a signalosome with Wnt ligand and Frizzled receptor to activate downstream cascade. The phosphorylation of PPSPXS motifs of LRP6 could recruit other Wnt signaling components Disheveled (Dvl), Axin and GSK3β to signalosome to amplify and stabilize its phosphorylation, which can directly inhibit GSK3β activity leading to multiple downstream cascades [12-14]. Therefore, LRP6 represents an important regulatory node in transducing Wnt stimulation. Recent reports reveal that LRP6 contributed to tumor progression in colorectal cancer and some other cancers [15-18]. However, the mechanism about LRP6 in colorectal cancers is seldom investigated.

In this study, we demonstrated that phosphorylated LRP6 was markedly up-regulate in colorectal carcinoma and indicated poor prognosis. The activation of LRP6 could enhance Wnt signaling and promote cell migration. LRP6 governed the cytoskeleton stability and remodeling through microtubule-related protein: microtubule-associated protein 1B (MAP1B) and microtubule actin cross-linking factor 1 (MACF1) as well as Rho GTPases.

## RESULTS

### Expression of Phosphorylated LRP6 is correlated with aggressive clinical behavior in colorectal carcinoma

**Figure 1:**
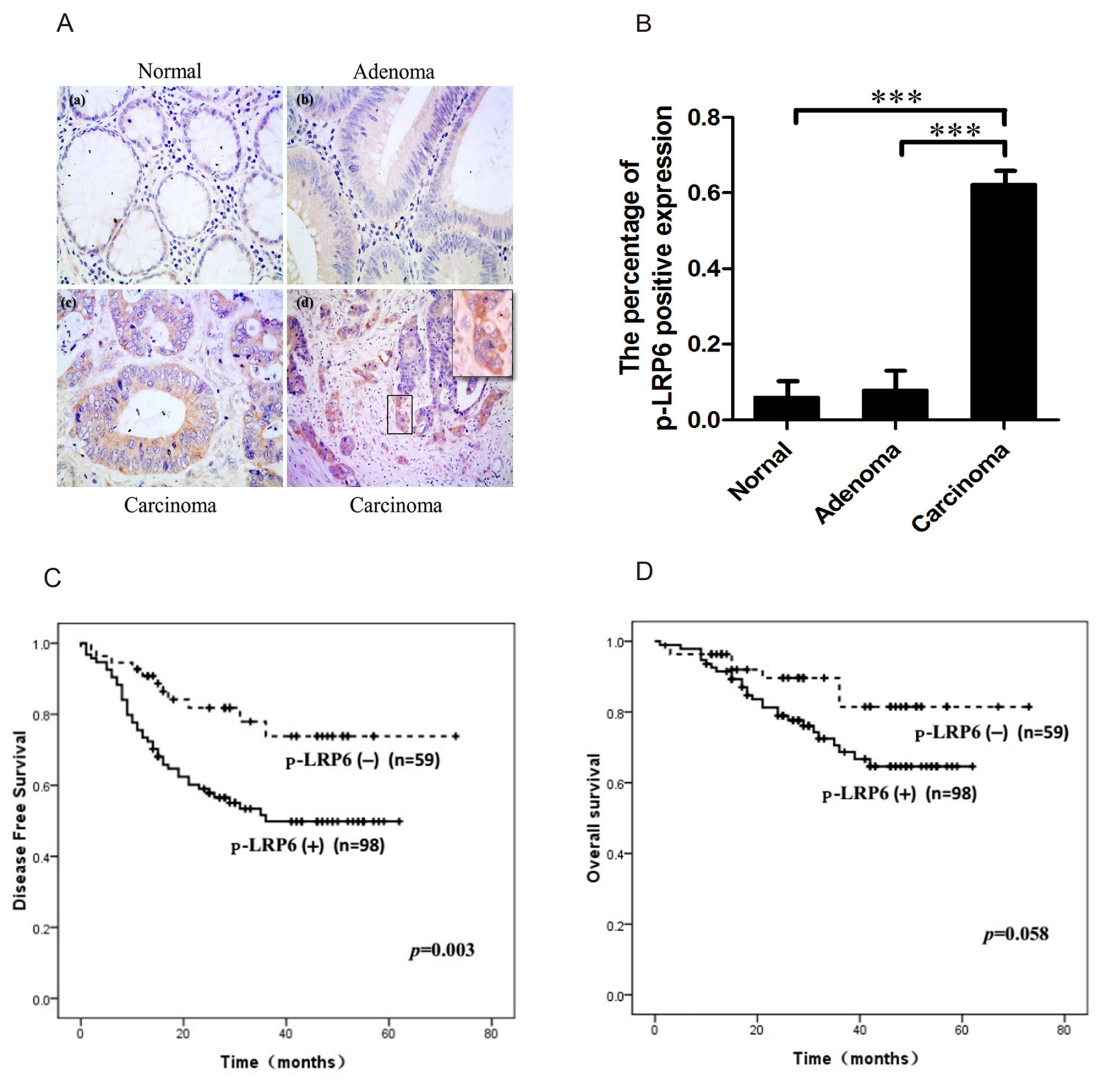
Immuno-staining of p-LRP6 correlated with outcome of colorectal cancers **(A)** The representative images of p-LRP6 staining in normal glandular cells (a), adenoma (b) and carcinoma (c) (×200). High magnification of p-LRP6 stained in front zone of cancer invasion (×800) (d). **(B)** The percentage of positive p-LRP6 expression in normal glandular cells, adenoma, and colorectal carcinoma. **(C)** Kaplan-Meier survival curves for the relation of p-LRP6 immuno-staining with DFS. **(D)** Kaplan-Meier survival curves for the relation of p-LRP6 immuno-staining with OS. ^*^ *p*< 0.05, ^**^p< 0.01, ^***^p< 0.001.

Immuno-staining of both LRP6 and phosphorylated LRP6 (p-LRP6) were performed in colorectal carcinoma tissue. The expression of LRP6 was negative in most cases (91.2%, 62/68) with few positive (8.8%, 6/68). However, the immunostaining of p-LRP6 was in a diffuse pattern in cytoplasm with no apparent membranous expression. Comparing to non-neoplastic glandular cells (4.8%, 1/21) and adenoma tissue (5.3%, 2/38), the expression of p-LRP6 was much more intense in carcinoma with higher positive level (62.3%, 114/183), which displayed the significant statistical difference (*p*<0.001) (Figure 1A, 1B). It was noteworthy that positive p-LRP6 was usually observed at the invasive front tumor zone in infiltrating nests of cancers (Figure 1A).

Wnt signaling plays a pivotal role in colorectal carcinogenesis and the nuclear accumulation of β-catenin is widely accepted as the hallmark of Wnt signaling activation [19, 20]. Accordingly, the expressions of β-catenin was immunohistochemically evaluated. Positive nuclear expression of β-catenin was 74.3% (136/183) in carcinoma, which was higher than adenoma tissue (55.3%, 21/38) (*p*<0.05) (Supplementary Figure 1). At the same time, the cytoplasm expression of β-catenin was also found more frequently in carcinoma cells (79.8%, 146/183) than adenoma (57.9%, 22/38) (*p*<0.05) (Supplementary Figure 1A, 1B). Besides, a positive correlation between p-LRP6 and nuclear expression of β-catenin was established (Supplementary Table 1).

The correlations between p-LRP6 expression and clinicopathologic parameters such as age, gender, tumor size, depth of invasion, lymph node metastasis, TNM and Dukes staging were analyzed (Supplementary Table 2). Positive p-LRP6 was found in 92 of 115 cases (80.0%) of advanced carcinoma (T3 and T4), while early carcinomas (T1 and T2) showed positive staining in just 35.3% (24/68) (*p*<0.001, χ^2^=36.801). 76.6% (59/77) of cases with lymph node metastasis was positive p-LRP6, but only 48.8% (39/80) (*p*<0.001, χ^2^=12.995) without metastasis showed p-LRP6 expression. In addition, there was a positive linear correlation between p-LRP6 expression and TNM or Dukes staging (*p*<0.001). Kaplan-Meier single-factor analysis and log-rank test demonstrated a statistically significant decrease in disease-free survival (DFS) in patients with positive staining of p-LRP6 (*p=*0.003) (Figure 1C). However, evaluation of patients’ overall survival (OS) displayed a borderline significance between positive and negative expression of p-LRP6 (*p=*0.058) (Figure 1D).

Taken together, these data proved that the p-LRP6 was involved in Wnt signaling activation and correlated with a more aggressive phenotype with higher metastatic potential and worse prognosis.

### Activation of LRP6 increases Wnt/β-catenin signaling in colorectal cancer cells

**Figure 2:**
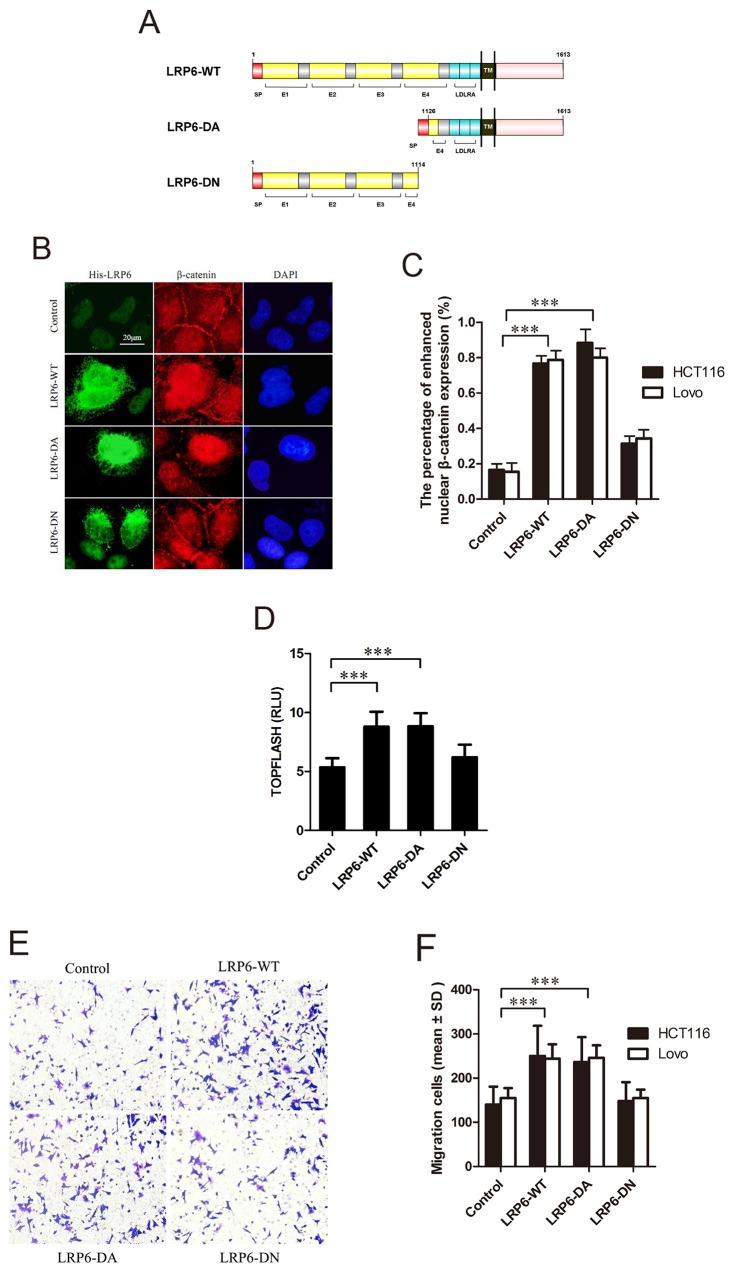
Activated LRP6 stimulates Wnt/β-catenin signaling and promotes cell migration **(A)** Schematic diagram of His-LRP6 deletion mutants. **(B)** The effects of LRP6 on nuclear translocation of β-catenin. Lovo and HCT116 cells were transfected with control vector, LRP6-WT, LRP6-DA and LRP6-DN and expression of His-tag and β-catenin were measured by immunofluorescent staining. The data showed was HCT116 cells. **(C)** The statistical analysis of β-catenin nuclear translocation in each group. **(D)** TOPFLASH activity upon different LRP6 expression on HCT116 cells. **(E)** Expression of different domains of LRP6 influenced migration of cancer cells. Lovo and HCT116 cells with transfection of control vector, LRP6-WT, LRP6-DA and LRP6-DN were subjected to Transwell analysis, respectively (×200). The data showed was from Lovo cells. **(F)** The number of cells counted in migration assay and the statistical analysis. Scale bar, 20 μm. Values represent mean ± SD of three experiments. ^*^ *p*< 0.05, ^**^p< 0.01, ^***^p< 0.001. All the experiments were repeated at least three times.

To evaluate the role of LRP6 in activation of Wnt signaling, the plasmids LRP6-WT (wild-type LRP6), LRP6-DA (dominant active, reserving the intracellular domain (ICD) of LRP6, residues 1126-1613) and LRP6-DN (dominant negative, reserving the extracellular domain of LRP6, residues 1-1114) were constructed (Figure 2A). With transfection of Lovo and HCT116 cells with above plasmids, β-catenin immunofluorescence staining and luciferase assay were carried out. The results showed that nuclear staining of β-catenin was obviously enhanced in LRP6-WT and LRP6-DA group with attenuated membrane expression comparing to control or LRP6-DN (*p*<0.001) (Figure 2B, 2C). Expectedly, overexpression of LRP6-WT and LRP6-DA led to an activation of TCF/β-catenin reporter up to about 2-fold, as compared to the vector control or LRP6-DN (*p*<0.001) (Figure 2D). The data demonstrated that activation of LRP6 could increase Wnt signaling activity in colorectal cancer cells.

### Activation of LRP6 promotes migration of colorectal cancer cells

To determine whether up-regulation of LRP6 would influence cancer cell migration and invasion, Transwell assays with or without Matrigel were performed. In migration assay, Lovo and HCT116 cells transfected with LRP6-WT or LRP6-DA plasmids demonstrated much higher motility potential than vector control group or LRP6-DN group (*p*<0.001) (Figure 2E, 2F). As for invasion assay, we failed to found the statistical difference between each group, however, the results showed the tendency of promoted invasive potential in LRP6-DA and LRP6-WT transfected cells (*p*=0.087) (Supplementary Figure 2).

Above evidence suggested the possible role of LRP6 in promoting the migration in colorectal tumor cells.

### Activation of LRP6 enhances cytoskeletal dynamics and remodeling

Phosphorylation or overexpression of LRP6 contributed to colorectal carcinoma migration and invasion, which arise the question about how LRP6 manage to control such aggressive behavior. It was widely agreed that migratory cancer cells undergo dramatic molecular and cellular changes by remodeling their cytoskeleton. Reorganization of the actin cytoskeleton and the concomitant formation of membrane protrusions required for cell motility, including focal adhesion, lamellipodia, filopodia, podosomes and invadopodia [21, 22]. On the other hand, polymerization of microtubules (MTs) also attribute to cancer cell migration and invasion. MT cytoskeleton is polarized in migrating cells and is essential for the directed migration [23]. We therefore analyzed the function of LRP6 in regulating cytoskeletal remodeling and stability that may responsible for colorectal carcinoma migration and invasion.

**Figure 3:**
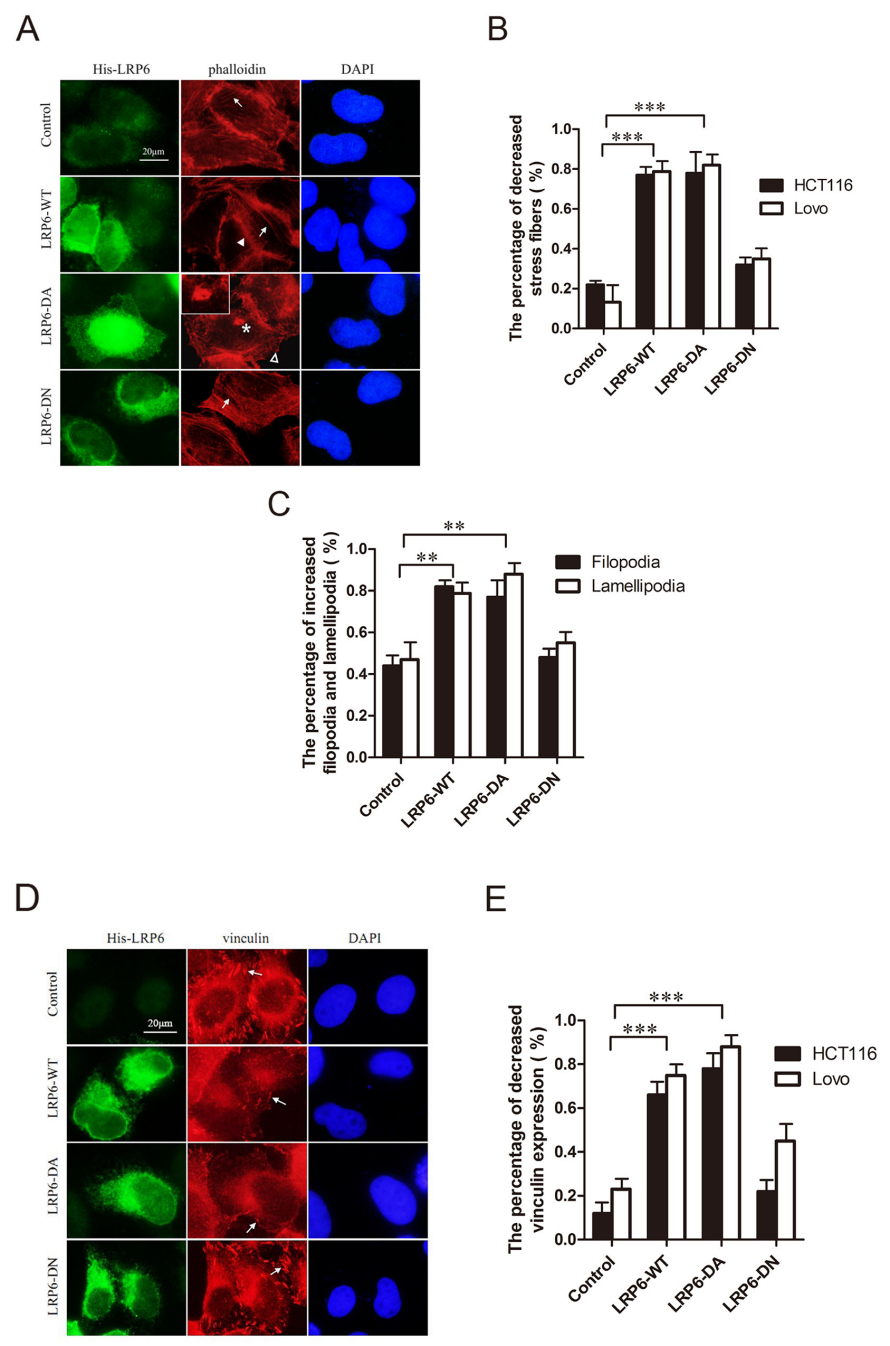
Activation of LRP6 increases actin remodeling **(A)** Overexpression of LRP6 induced actin remodeling. Lovo and HCT116 cells were transfected by control vector, LRP6-WT, LRP6-DA and LRP6-DN and doubly stained by His and phalloidin. Arrows indicate stress fibers, asterisk indicate the formation of invadopodia, arrowhead indicate filopodia and hollow arrowhead indicate lamellipodia. The data showed was from HCT116 cells. **(B)** The percentage of decreased stress fibers in each group with statistical analysis. **(C)** The percentage of increased filopodia and lamellipodia in each group with statistical analysis on HCT116 cells. **(D)** Overexpression of LRP6 reduced focal adhesions of cells. Lovo and HCT116 cells were transfected by control vector, LRP6-WT, LRP6-DA and LRP6-DN and doubly stained by His and vinculin. The arrows indicate focal adhesions. The data showed was from HCT116 cells. **(E)** The percentage of decreased vinculin expression in each group with statistical analysis. Scale bar, 20 μm. Values represent mean ± SD of three experiments. ^*^ *p*< 0.05, ^**^p< 0.01, ^***^p< 0.001. All the experiments were repeated at least three times.

F-actin and focal adhesions, labeled respectively by phalloidin and vinculin, were observed under immunofluorescence staining. With up-regulated LRP6-WT and LRP6-DA, Lovo and HCT116 cells stained by phalloidin demonstrated a decrease in stress fibers, while cell membrane protrusions such as filopodia or lamellipodia increased with emergence of invadopodia in a portion of cells in LRP6-DA group (Figure 3A, 3B, 3C). At the same time, focal adhesions labeled with vinculin decreased (Figure 3D, 3E). Actin skeleton seemed to be rearranged upon LRP6 overexpression and enabled tumor cells to gain increased migratory capability.

**Figure 4:**
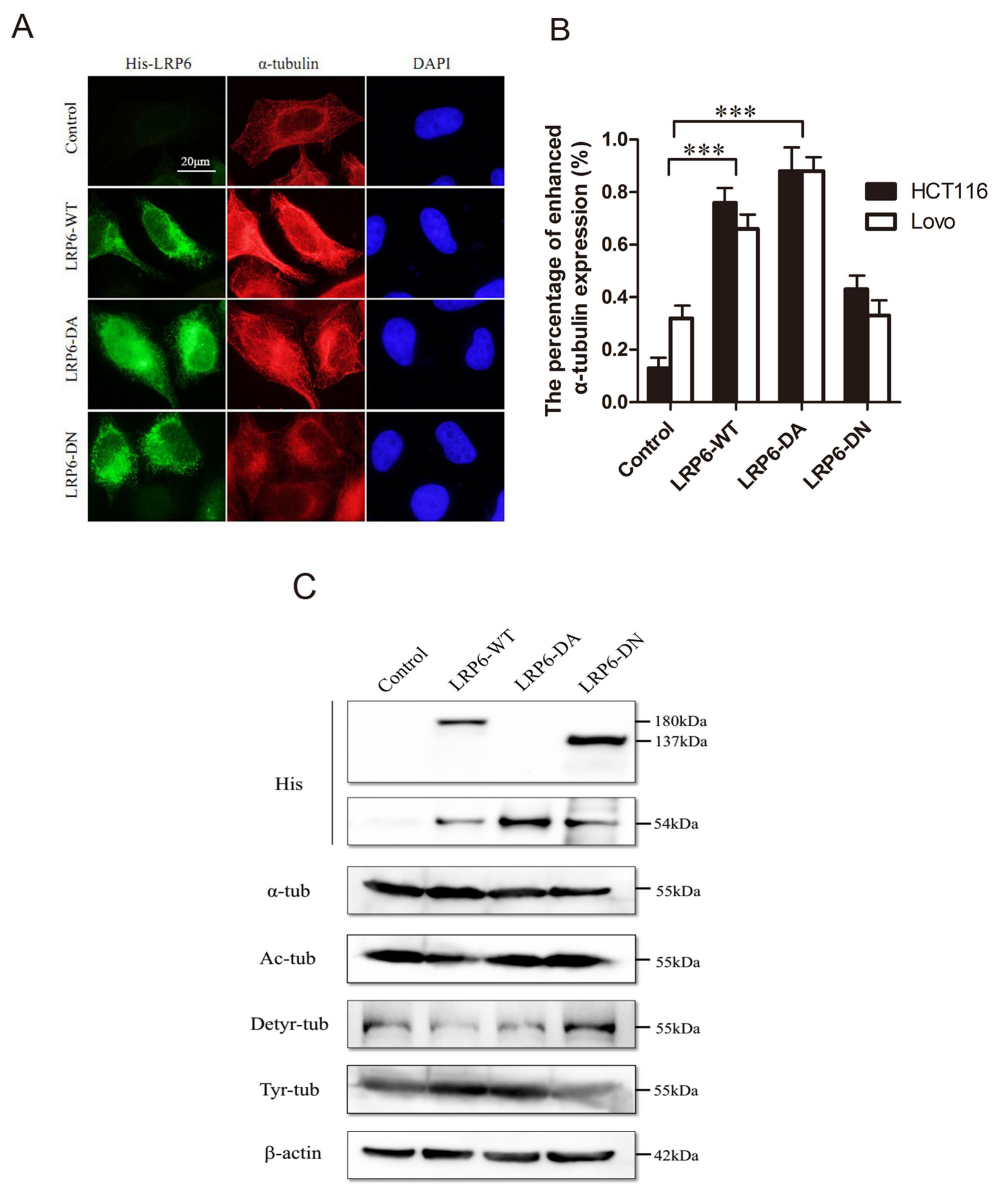
Activation of LRP6 increases MT dynamics and post-modification **(A)** Overexpression of LRP6 increase cellular ɑ-tubulin. Lovo and HCT116 cells were transfected by control vector, LRP6-WT, LRP6-DA and LRP6-DN and then stained by His and ɑ-tubulin. The data showed was from HCT116. **(B)** The percentage of increased ɑ-tubulin expression in each group with statistical analysis. **(C)** The effect of LRP6 expression on level of Acetyl-ɑ-tubulin, detyrosinated-ɑ-tubulin, tyrosine-ɑ-tubulin in tumor cells. Lovo and HCT116 cells were transfected with different LRP6 plasmids and cell extracts were prepared and analyzed by Western Blotting. The data showed was from Lovo. Scale bar, 20 μm. Values represent mean ± SD of three experiments. ^*^ *p*< 0.05, ^**^p< 0.01, ^***^p< 0.001. All of experiments were repeated at least three times.

The acetylation and detyrosination/retyrosination of MTs acted as significant post-translational modifications. The acetylation and detryrosination modification have commonly been linked to MT stability [24, 25] while the retyrosination is generally considered to occur on dynamic MT assemblies [25]. As for MTs, the accumulation of LRP6 could cause an increase in intensity of α-tubulin staining (Figure 4A, 4B). In addition, upon transfection with LRP6-WT and LRP6-DA, acetylated tubulins and detyrosinated tubulins were decreased, while tyrosinated-tubulins were obviously increased (Figure 4C). Thus, it appeared that overexpression of LRP6 stimulated the overall dynamics of MT network.

### The involvement of MAP1B and MACF1 in LRP6 modulation on MT cytoskeleton

**Figure 5:**
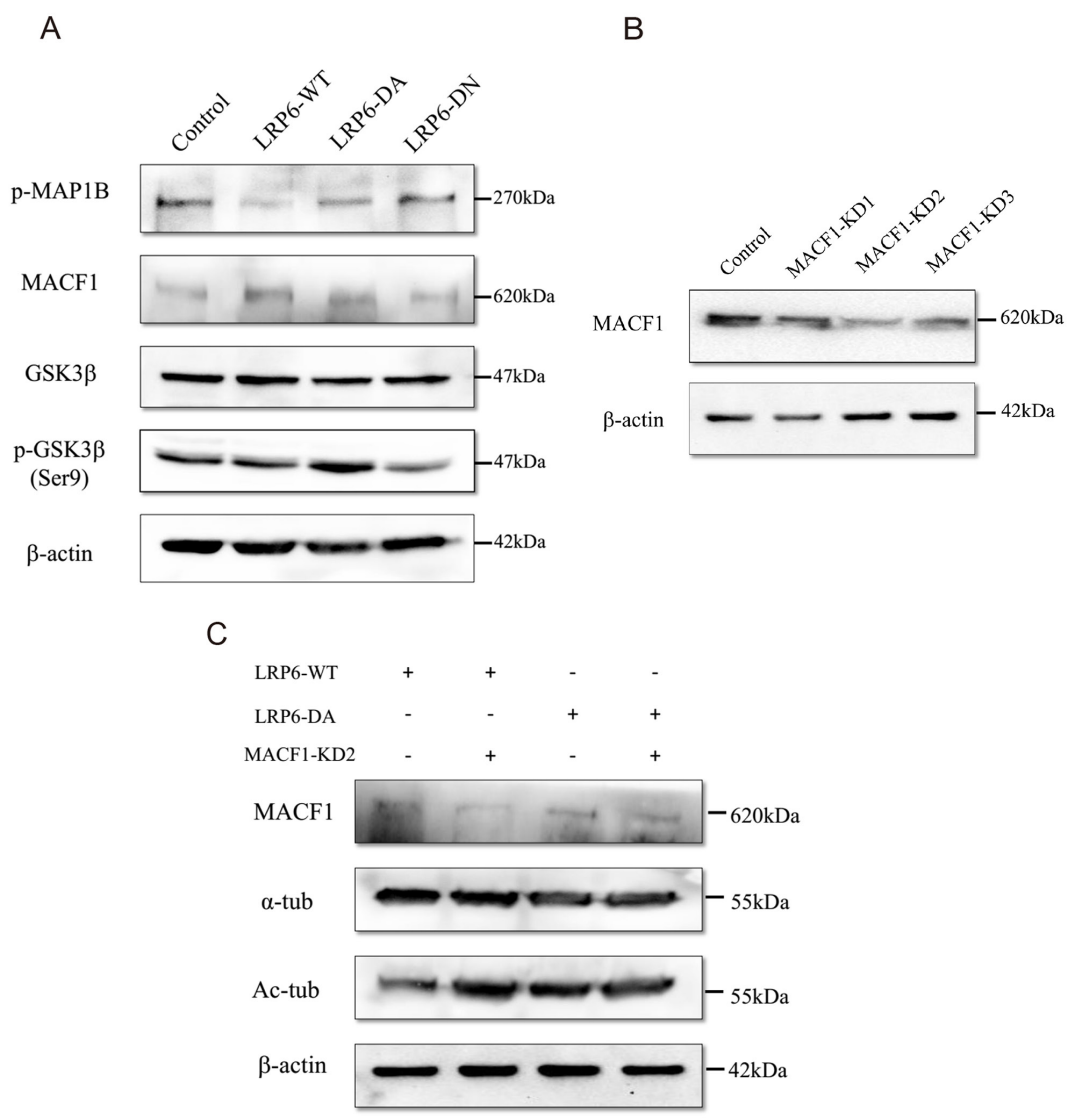
The underlying relationship between LRP6 and MAP1B, MACF1 and GSK3β **(A)** Lovo and HCT116 cells were treated with control vector, LRP6-WT, LRP6-DA and LRP6-DN and cell extracts were prepared and analyzed by Western Blotting. The protein level of phosphor-MAP1B, MACF1, total-GSK3β and phosphor-GSK3β (Ser9) were evaluated. The data showed was from Lovo. **(B)** Depletion of MACF1 with intervening RNA. Lovo cells were transfected with MACF1 RNAi: MACF1-KD1, -KD2, -KD3 or control, and levels of MACF1 were measured by Western blotting. **(C)** Knockdown of MACF1 stabilized MTs. Lovo cells were co-transfected with MACF1 RNAi and LRP6-WT or LRP6-DA and protein level analyzed by Western Blotting. All of experiments were repeated at least three times.

Previous evidences suggests that MT assembly and stability are highly regulated by microtubule-associated proteins (MAPs) [26]. MAP1B is one of the most significant members of MAPs and acts to maintain the stability of MT by regulating the local balance among acetylated, detyrosinated and tyrosinated MTs [27]. The function of MAP1B is controlled by phosphorylation [28], which is modulated by GSK3β [27, 29]. To further demonstrate how LRP6 modulates the dynamic process of MT assembly, we evaluated the status of phosphorylation of MAP1B (p-MAP1B) and GSK3β in Lovo and HCT116 cells. Up-regulation of LRP6 through transfection of LRP6-WT and LRP6-DA resulted in the attenuated level of p-MAP1B (Figure 5A). Consistently, the inactivated form of GSK3β: phosphor-GSK3β (Ser9) was obviously increased in LRP6-DA group while the total level of GSK3β remained roughly unchanged (Figure 5A). Our data suggested that LRP6 may negatively regulate the phosphorylation of MAP1B through suppression of GSK3β.

Another factor included in our study that regulated MT cytoskeleton was MACF1--a multidomain protein that can promote MT dynamics to assist cell migration [30]. In this study, overexpression of MACF1 was observed upon LRP6 overexpression (Figure 5A) in Lovo and HCT116 cells. After knockdown of MACF1 by interfering RNA (Figure 5B), acetylated tubulins were increased in LRP6-WT and LRP6-DA group (Figure 5C), which indicated that MACF1 was responsible for the MT instability. These data could likely be elucidated that LRP6 modulated MT assembly through upregulating MACF1 level.

### LRP6 regulates actin remodeling via Rho GTPase

Members of the RhoGTPse family play a pivotal role in transmitting signals from upstream regulatory molecular to effector proteins of actin cytoskeleton remodeling. RhoGTPases are activated upon GTP binding and inactive in their GDP-bound form [31]. RhoA, Rac1, and Cdc42 are the best studied members of the RhoGTPase family and their critical role in cell migration and invasion via actin remodeling has been extensively described [32, 33]. To address the function of LRP6 in actin remodeling, we analyzed the association of LRP6 with Rho GTPase members: RhoA, Rac1 and Cdc42.

**Figure 6:**
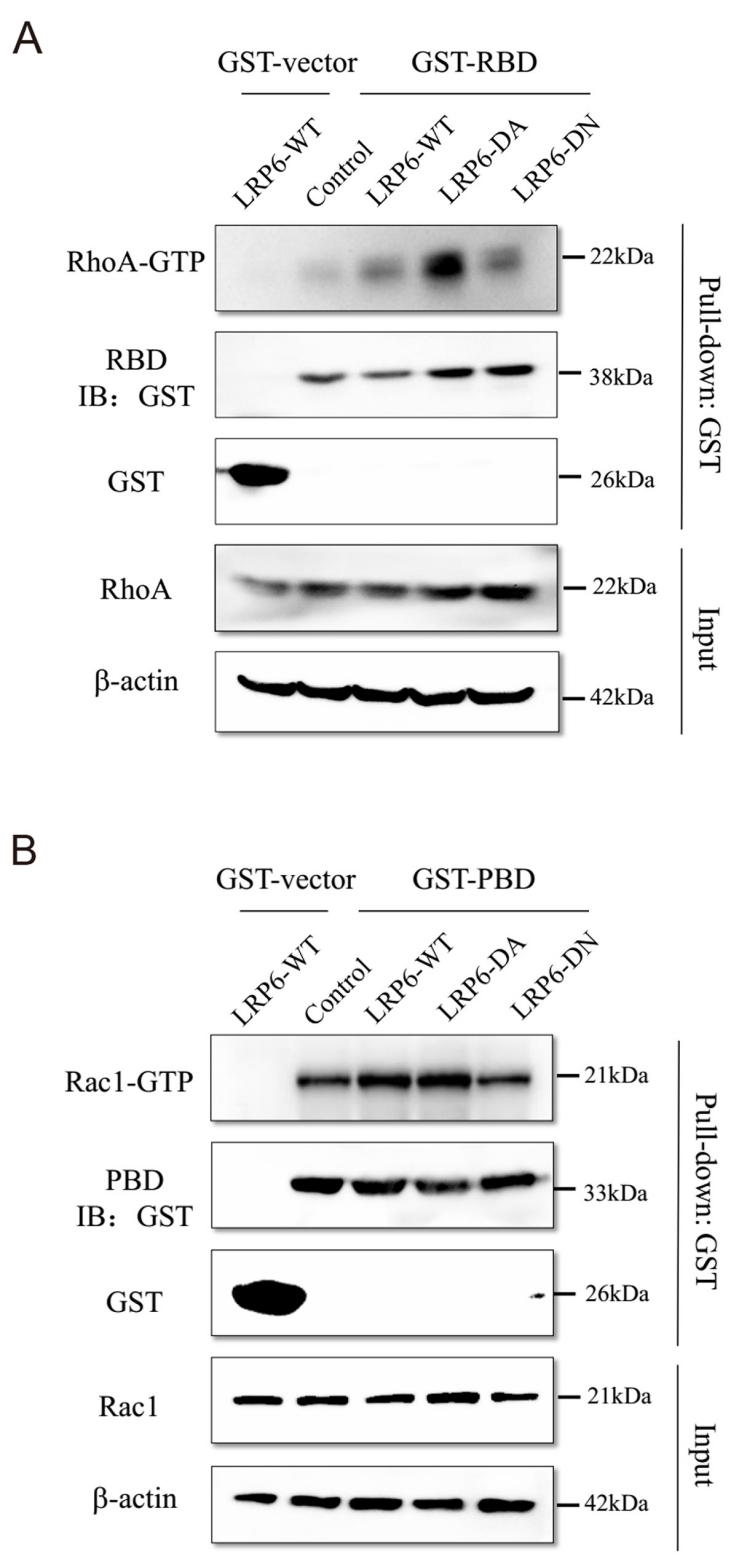
LRP6 regulate Rho GTPase activity **(A)** Up-regulation of LRP6 level increased the active GTP-bound RhoA. The GST fusion protein of RBD were expressed in BL21 cells and purified with glutathione agaroses. Lovo and HCT116 cells were transfected with control vector, LRP6-WT, LRP6-DA and LRP6-DN and cell extracts were mixed with the produced agaroses. Then, the level of RhoA in GST pull-down lysate was evaluated by Western Blotting. **(B)** Up-regulation of LRP6 level increased the active GTP-bound Rac1, the GST fusion protein of PBD were expressed in BL21 cells and purified with glutathione agaroses. Lovo and HCT116 cells were transfected with control vector, LRP6-WT, LRP6-DA and LRP6-DN and cell extracts were mixed with the produced agaroses. Then, the level of Rac1 in GST pull-down lysate was evaluated by Western Blotting. All data showed was from HCT116. All the experiments were repeated at least three times.

Active GTP-bound form of RhoA, Rac1 and Cdc42 were evaluated in different LRP6 truncation transfected cells by GST pull-down assay. In GST pull-down assay, we purified the GST fusion protein of GST vector, GST-PBD, GST-RBD and GST-WASP which were the adapter protein of active GTP-bound RhoA, Rac1 and Cdc42 (Supplementary Figure 3). The date showed that form of RhoA-GTP was markedly enhanced in LRP6-DA group comparing to LRP6-WT and LRP6-DN group (Figure 6A). As for Rac1-GTP, LRP6-WT and LRP6-DA transfection displayed up-regulatory effect on Rac1-GTP than LRP6-DN (Figure 6B). Whereas Cdc42-GTP didn’t show the same trend as RhoA and Rac1 (Supplementary Figure 4). It was likely that activate RhoA and Rac1, but not Cdc42 undergone regulation of LRP6, especially through its intracellular domain, to carry out the actin remodeling function.

## DISCUSSION

The vast of evidences have been accumulated that Wnt signaling is involved in progression of colorectal cancers, through either inducing epithelial–mesenchymal transition (EMT) [8], inhibiting tumor cell apoptosis [34] or increasing tumor angiogenesis [35]. The resultant phenotypes in activation of Wnt signaling have been credited to the nuclear translocation of β-catenin, which triggers a series of target genes for mediating cellular biological activities. Recent studies have reveal an important role of LRP6 in contributing to tumor progression in breast cancer, hepatocellular carcinoma and colorectal cancer [36], but the exact molecular mechanism is still elusive.

The cell process in EMT, apoptosis andangiogenesis involves not only transcription of genes, but also cytoskeleton dynamics. Several lines of evidence have suggested the components of Wnt signaling functioned in modulating cytoskeleton remodeling. For instance, Dvl could directly binding to MTs and enhance the stability and it can also modulate actin cytoskeleton remodeling through DAMM1/RhoA axis and Rac1 axis [37]. More important, GSK3β could directly phosphorylate MAPs to increase MTs polymerization [38]. Axin acts as a component of Wnt signaling in translocation of Axin and MACF1 associated complex from the cytoplasm to the cell membrane [39]. Therefore, the three members of Dvl, Axin and GSK3β of Wnt signalosome could regulate cytoskeleton dynamics, respectively. LRP6 is one of the co-receptors of Wnt pathway and phosphorylation of LRP6 could recruit Dvl, Axin and GSK3β to signalosome that leading to multiple downstream cascades [12-14] to regulate cytoskeleton activities. LRP6 is a multidomain transmembrane protein and its ICD is indispensable for downstream signaling. It contains an S/T cluster and five PPSPXS motifs that could be phosphorylated by several cytoplasmic kinases according to different physical circumstance [13]. Phosphorylated PPSPXS motifs recruits Axin and GSK-3β to bind directly to ICD and LRP6 ICD directly inhibits GSK-3β phosphorylation of β-catenin [13, 14].

As showed in Figure 5A, the expression of LRP6-WT or LRP6-ICD could decrease MAP1B phosphorylation, which may result in decreasing of acetylated, detyrosinated MTs and increasing of tyrosinated MTs in colorectal cancer cells. It could be that elevated expression of LRP6-WT or LRP6-ICD could directly inhibit GSK-3β activity through binding, which decreased MAP1B phosphorylation. In addition, the expression status of LRP6 also altered inhibitory phosphorylation of GSK-3β (Figure 5A), indicating that activation of LRP6 may enhance GSK-3β phosphorylation, which has been catalyzed by AKT1. Actually, we have found that expression of LRP6 could stimulate PI3K/AKT pathway (unpublished data).

MACF1 is a cross-link factor between MTs and actins with ability to bridge MT and actin cytoskeletal networks to assist cell migration [30]. Research has showed that MACF1 could bind with Axin to form a complex in translocation from cytoplasm to cell membrane, acting as a component of Wnt signaling [39]. While the activation of LRP6 could recruit Axin [40] and resultantly increase its bind to MACF1.

Apparently, it is reasonable that LRP6 could be involved in the regulation of cytoskeleton dynamics, as showed in this described investigation. As far as we know, this study was the first report investigating the role of LRP6 in cytoskeleton dynamics.

Actin remodeling is indispensable for invasive protrusions in cell migration. The cytoskeleton remodeling is generally regulated by well-known Rho GTPases which includes three members of RhoA, Rac1, and Cdc42 [32, 33]. In the present investigation, LRP6 overexpression led to a decrease in stress fibers and focal adhesion but an increase in cell membrane protrusions. In this study, we revealed that the active form of LRP6, LRP6-ICD, obviously upregulated level of active RhoA and Rac1. Although RhoA was reported to induce stress fiber formation and promotes cytoskeletal configurations affecting cell-cell or cell-matrix adhesion [31], some studies have shown that RhoA has dynamic function in tumor cell migration and invasion through suppressing stress fiber generation to permit RhoA mediated lamellipodia formation [41]. And apparently, Rac1 could induce formation of cellular protrusions such as lamellipodia and membrane ruffle for mediating cell motility [42-44]. However, how LRP6 regulate RhoGTPase activity has been little documented. A recent study reveals that LRP6 status could influence RhoA activity and also could directly interact with DAAM1 (Disheveled-associated activator of morphogenesis 1), a formin promoting GEF activity in Wnt signaling [45]. In addition, some investigations imply that GSK3β could regulate RhoA and Rac1 [46, 47] and Dvl promote RhoA-Rac1 signaling [37] and non-classic Wnt/Planar cell polarity (PCP) signaling has been identified to be able to trigger activation of the small GTPases RhoA and Rac1, leading to actin polymerization. Thus, it seems that Wnt signaling could regulate actin remodeling in many aspects.

In conclusion, we found active form of LRP6--phosphorylated LRP6 was markedly up-regulate and indicated poor prognosis in colorectal carcinoma. We investigated the mechanism of LRP6 on invasion and metastasis and found that LRP6 regulated cytoskeleton remodeling via MAP1B, MACF1 and Rho GTPase to promote metastasis. Further research efforts in identifying potential drug targets to disrupt LRP6 cytoskeleton modulation could provide a novel therapeutic strategy to improve metastatic colorectal cancer treatment.

## MATERIALS AND METHODS

### Ethics statement

This study was approved by the Peking University Institutional Review Board and Ethics Committee prior to the start of the project.

### Patients and tissue specimens

A total of 183 surgically resected cases of colorectal carcinoma were collected from the archives of the Department of pathology, Peking University Health Science Center. The patients included 100 males and 83 females, with a median age of 66.4 years (age range from 27 to 91 years). None of the patients received preoperative chemotherapy. 4% buffered formalin fixed and paraffin-embedded sections (4μm thick) were stained with hematoxylin and eosin for histologic evaluation of histological diagnosis, typing and grading. Age, gender, tumor size, depth of invasion, lymph node metastasis, TNM stage and Dukes stage were obtained by review of medical charts and pathologic records. The immunostaining of DNA mismatch repair genes (MMR), including MLH1, MSH2 and MSH6, was positive in all of 183 cases, indicating their belonging to sporadic colorectal carcinoma.

Among the patients, 157 cases were followed to determine postoperative survival. During follow-up, tumors recurred in 43 patients (27.4%), and sites of recurrence included liver (25 patients), lung (6), lymph nodes (3), and peritoneum (2), colon (2), ovary and pelvis (2), bone (2), brain (1). 32 patients (20.4%) were dead of disease by the end of the follow-up period.

### Antibodies

The mouse monoclonal anti-LRP6 antibody was from Santa Cruz (Santa Cruz, CA, USA). Rabbit polyclonal anti-p-LRP6 (Ser1490) was from Bioss (Woburn, MA, USA). Mouse monoclonal anti-β-catenin was from BD (Franklin Lakes, NJ, USA). Anti-phalloidin was from Life (Carlsbad, CA, USA). Mouse monoclonal anti-vinculin was from Boster (Wuhan, China). Mouse monoclonal anti-α-tubulin, acetyl-α-tubulin, tyrosine-α-tubulin were from Sigma-Aldrich (Saint Louis, MO, USA). Rabbit polyclonal anti-detyrosinated-α-tubulin, Mouse monoclonal anti-RhoA, Rac1and Cdc42 were purchased from Abcam (Cambridge, MA, USA). Rabbit polyclonal anti-phosphor-MAP1B (Thr1265) was from Novusbio (Littleton, CO, USA). Rabbit polyclonal anti-MACF1 was from Proteintech (Chicago, IL, USA). Rabbit monoclonal anti-phosphor-GSK3β (Ser9) and mouse monoclonal anti-GSK3β were from Cell Signaling (Beverly, MA, USA). Rabbit polyclonal anti-His, mouse monoclonal anti-His and anti-GST were from Origene (Rockville, MD, USA).

### Immunohistochemical staining

The immuno-staining was performed as described previously [48]. Staining was individually evaluated by two observers (QY and WH) blinded to all clinicopathological information. Discrepancies in analysis were reconciled following review by a third clinician (BZ). Expression of p-LRP6 presented in cytoplasm, membrane and combined, and ≥10% cells with cytoplasma staining of p-LRP6 was regarded as positive. β-catenin staining was found to be membranous, cytoplasmic, nuclear, or some combination of these, and ≥10% cells with β-catenin staining were regarded as positive.

### Cell culture

Hela, Lovo and HCT116 cells were cultured in Dulbecco modified Eagle medium with high glucose supplemented with 10% fetal bovine serum (Gibco, Life Technologies). Cells were incubated in a humidified atmosphere with 5% CO_2_ at 37 °C.

### Plasmid construction and transfection

The wild-type his-LRP6 pOTENT-1 (LRP6-WT) was from YouBio biotechnology company (Wuhan, China). It was digested with BglII (NEB), then re-ligated to form the truncated his-LRP6 with ICD, which regarded as the constitutive active form of LRP6 (LRP6-DA, reserving LRP6-ICD, residues 1126-1613) [49]. LRP6-WT was also digested with XhoI, BglII and EcoRI (NEB), end-filled and re-ligated to form the mutation only retain extracellular domain: 4 EGF-like repeats (LRP6-DN, deleting LRP6-ICD, residues 1-1114). TOPFLASH reporter construct and pRL-TK-Renilla Vector were purchased from Promega (Madison, USA). GST-PBD, GST-RBD and GST-WASP were constructed from fragment of PBD, RBD and WASP, which were amplified respectively from total cDNA using three pairs of primers (sense primer: 5’-CACAAGCTTAAAG AGCGGCCAGAG-3’ and antisense primer: 5’- CGACGCGTGTTGTAAA ACTCC AACACATC-3’ for PBD; sense primer: 5’- CACAAGCTTATCCTGGAGGACCTGAATATG-3’ and antisense primer: 5’- CGACGCGTGAGGTCAGAGATGCAGACCC-3’ for RBD; sense primer: 5’- CACAAGCTT GGACATCCAG AACCCTGACAT-3’ and antisense primer: 5’- CAGTGGACCAGAACGACCCTTGTTA-3’ for WASP), and were cloned into pEX-N-GST vector. The small interfering RNAs were designed and synthesized as following: MACF1-KD1, 5’-GCUGGUCACCUUGC GUCUATT-3’; MACF1-KD2, 5’-GCUCGUGACAU AAUGGAAATT-3’; MACF1-KD3, 5’-GCUCAUAGCCA AUCAGAAATT-3’; control, 5’-UUCUCCGAACGUGUC ACGUTT-3’. Plasmids were transfected with Lipofectamine 2000 (Invitrogen) and synthesized siRNAs with Lipofectamine RNAiMAX (Invitrogen) according to the manufacturer’s instructions.

### Immunofluorescent microscopy

Cells seeding, fixing and staining were performed as described previously [48]. The antibodies, including anti-β-catenin (1:2000), anti-phalloidin (1:200), anti-vinculin (1:100) and anti-α-tubuiln (1:500), were used, respectively. Images were observed and recorded using a fluorescence microscope (Model CX51; Olympus, Tokyo, Japan), and Photoshop version 7.0 (Adobe Systems Inc.) was used for image processing. The experiments were performed independently at least three times.

### Luciferase assay

Luciferase activity was examined using Luciferase Assay kit (Promega). In brief, after transfection of TOPFLASH reporter gene together with LRP6 mutantsin 24–well plate (8×10^4^ cells per well) for 48 h, lysates of Lovo and HCT116 cells were measured according to the assay kit protocol. All data were normalized by Renilla activity. The experiments were performed in duplicate and repeated independently at least three times.

### Transwell assay

Cells seeding, transfection, Matrigel preparation, fixing and staining were performed according to described previously [48]. Briefly, cells seeded on 24-well plate were transfected, and after 48 h, the cells (1×10^5^ cells) were seeded into the upper chamber of Transwell 24-well plates. For invasion assay, each insert was coated with 2 mg/ml Matrigel and incubated at 37°C for 1h. A total of 2×10^5^ cells were suspended in 100 ml serum-free DMEM media and loaded into coated inserts. Migration and invasion chambers were incubated in a humidified 5% CO^2^ incubator at 37°C for 12h and 24h respectively. The experiments were repeated independently at least three times.

### Western blotting

The preparation of cells extracts, 10% SDS-PAGE, electronic transfer, and chemiluminescence were performed as described previously [48]. The antibodies were used including anti-acetyl-α-tubulin (1:1000), anti-detyrosinated-α-tubulin (1:500), anti-tyrosine-α-tubulin (1:1000), anti-His (1:1000), anti-β-actin (1:1000), anti-phosphor-MAP1B (1:1000), anti-MACF1 (1:500), anti-GSK-3β (1:5000), anti-GSK3β-S9 (1:1000), anti-RhoA (1:1000), anti-Rac1(1:2000), anti-Cdc42 (1:100), anti-GST (1:5000). The experiments were repeated independently at least three times.

### GST pull-down assay

Plasmids encoding GST and GST-tagged recombinant PBD, RBD and WASP domain proteins were expressed in Escherichia coli BL21 cells (Invitrogen), which was induced by IPTG for 3 hours at 30°C. Bacteria were lysed in the buffer PBS-L (50 mM NaH2PO4, 150 mM NaCl, pH 7.2, 1 mM DTT, 1 mM EDTA, 1% Triton X-100, 1 mg/ml Lysozyme and protease inhibitors (Roche)) and sonicated. The cytoplasmic fraction of bacterial lysates were mixed with Glutathione HiCap matrix (Qiagen) and rocked for 30 minutes at 4 °C, then eluted in the buffer PBS-EW (50 mM NaH2PO4, 150 mM NaCl, pH 7.2, 1 mM DTT, 1 mM EDTA). Purity of the protein was analyzed by SDS-PAGE and assessed by Coomassie Blue G-250 staining (Sigma-Aldrich). Equal amounts of immobilized GST fusion proteins were mixed with prepared cell lysates (control vector, LRP6-WT, LRP6-DA and LRP6-DN transfected cells) and incubated for 3 hours at 4 °C. The beads were eluted with PBS-EW for 3 times and the supernatants were collected and analyzed by Western blotting analysis. The experiments were repeated independently at least three times.

### Statistical analysis

All analysis was performed using SPSS statistics software (Version 17.0, Chicago, IL, USA). Relationships between tumor markers and other parameters were studied using the chi-square test, Fisher’s exact test, Continuity Correlation or the independent *t*-test when appropriate. The influence of LRP6 expression on patient prognosis was analyzed based on overall survival (OS) and disease-free survival (DFS). OS was defined as the time from initial diagnosis to death from any cause or last follow-up. DFS was estimated as the time from initial diagnosis to progression, recurrence, death or last follow-up. Both DFS and OS curves were plotted using the Kaplan-Meier method and compared with log-rank tests. A *p* value of less than 0.05 was considered to be of statistical significance. All the statistical tests and *p* values were two-sided, and the level of significance was set at <0.05 (^*^), <0.01 (^**^), or <0.001 (^***^)

## SUPPLEMENTARY MATERIALS FIGURES AND TABLES


