# Correction: LRP6 promotes invasion and metastasis of colorectal cancer through cytoskeleton dynamics

**DOI:** 10.18632/oncotarget.27710

**Published:** 2020-08-11

**Authors:** Qian Yao, Yu An, Wei Hou, Ya-Nan Cao, Meng-Fei Yao, Ning-Ning Ma, Lin Hou, Hong Zhang, Hai-Jing Liu, Bo Zhang

**Affiliations:** ^1^ Department of Pathology, School of Basic Medical Sciences, Peking University Health Science Center, Beijing 100191, China; ^2^ Department of Pathology, Beijing Tongren Hospital, Capital Medical University, Beijing 100730, China

**This article has been corrected:** The Funding information has been updated as follows:

## FUNDING

This project was supported by the National Natural Science Foundation of China (No. 81101998, No. 81372292 and No. 81502493).

Original article: Oncotarget. 2017; 8:109632–109645. 109632-109645. https://doi.org/10.18632/oncotarget.22759

