# Supplementary material for: LRP6 promotes invasion and metastasis of colorectal cancer through cytoskeleton dynamics
Source: Oncotarget. 2017 Nov 30;8(65):109632–45. doi: 10.18632/oncotarget.22759 (PMC5752548; doi:10.18632/oncotarget.22759)
Supplement: Supplementary file 1 [file oncotarget-08-109632-s001.pdf]

LRP6 promotes invasion and metastasis of colorectal cancer through cytoskeleton dynamics

SUPPLEMENTARY MATERIALS

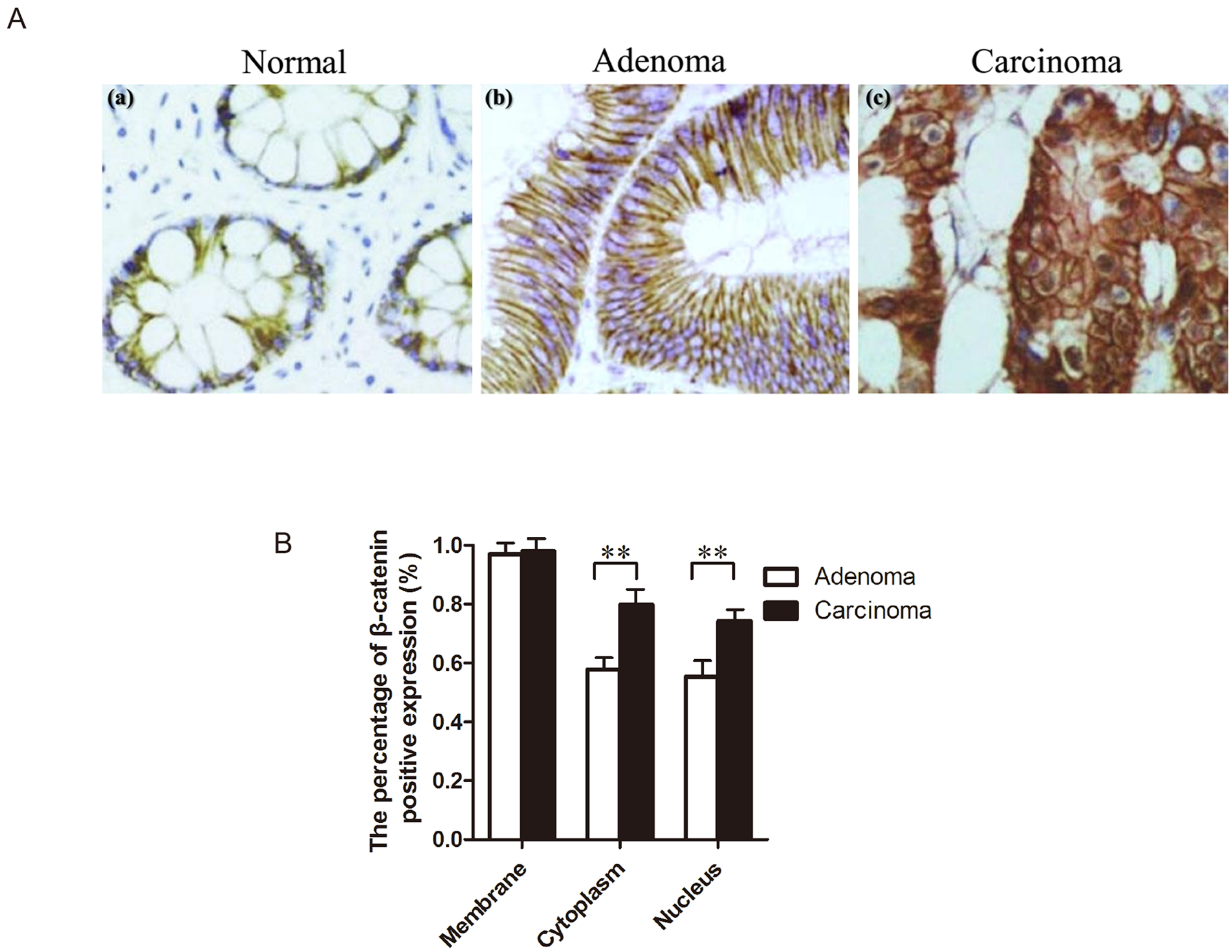

Supplementary Figure 1: The expression of β-catenin in colorectal tissue. (A) The representative images of β-catenin staining in normal glandular cells (a), adenoma (b) and carcinoma (c) (×200). **(B)** The subcellular β-catenin staining and positive percentage in normal glandular cells, adenoma and carcinoma. $^{*}p< 0.05$, $^{**}p< 0.01$, $^{***}p< 0.001$.

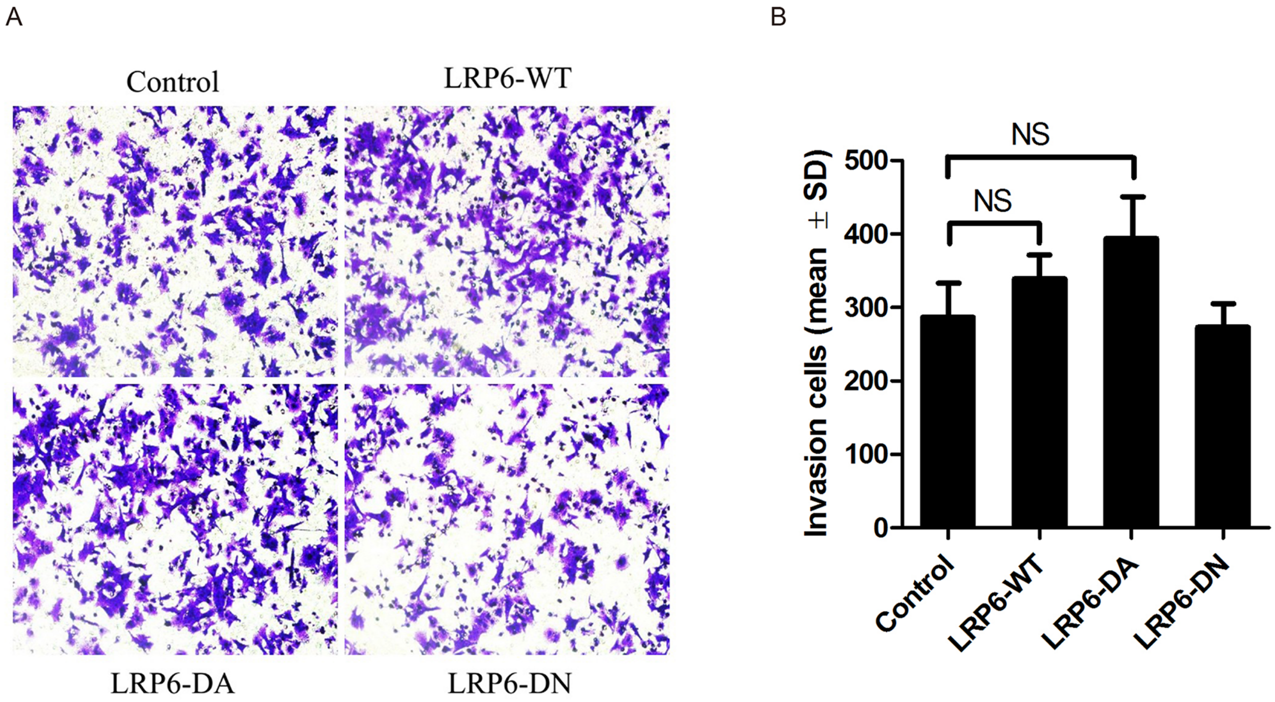

Supplementary Figure 2: The relationship between LRP6 overexpression and cell invasion in colorectal cancer. **(A)** The expression of different domains of LRP6 influenced invasion of cancer cells. Lovo and HCT116 cells with transfection of control vector, LRP6-WT, LRP6-DA and LRP6-DN were subjected to Transwell analysis, respectively (×200). **(B)** The number of cells counted in invasion assay and the statistical analysis. The data showed was from Lovo. Values represent mean ± SD of three experiments. $^{*}p< 0.05$, $^{**}p< 0.01$, $^{***}p< 0.001$. All of experiments were repeated at least three times.

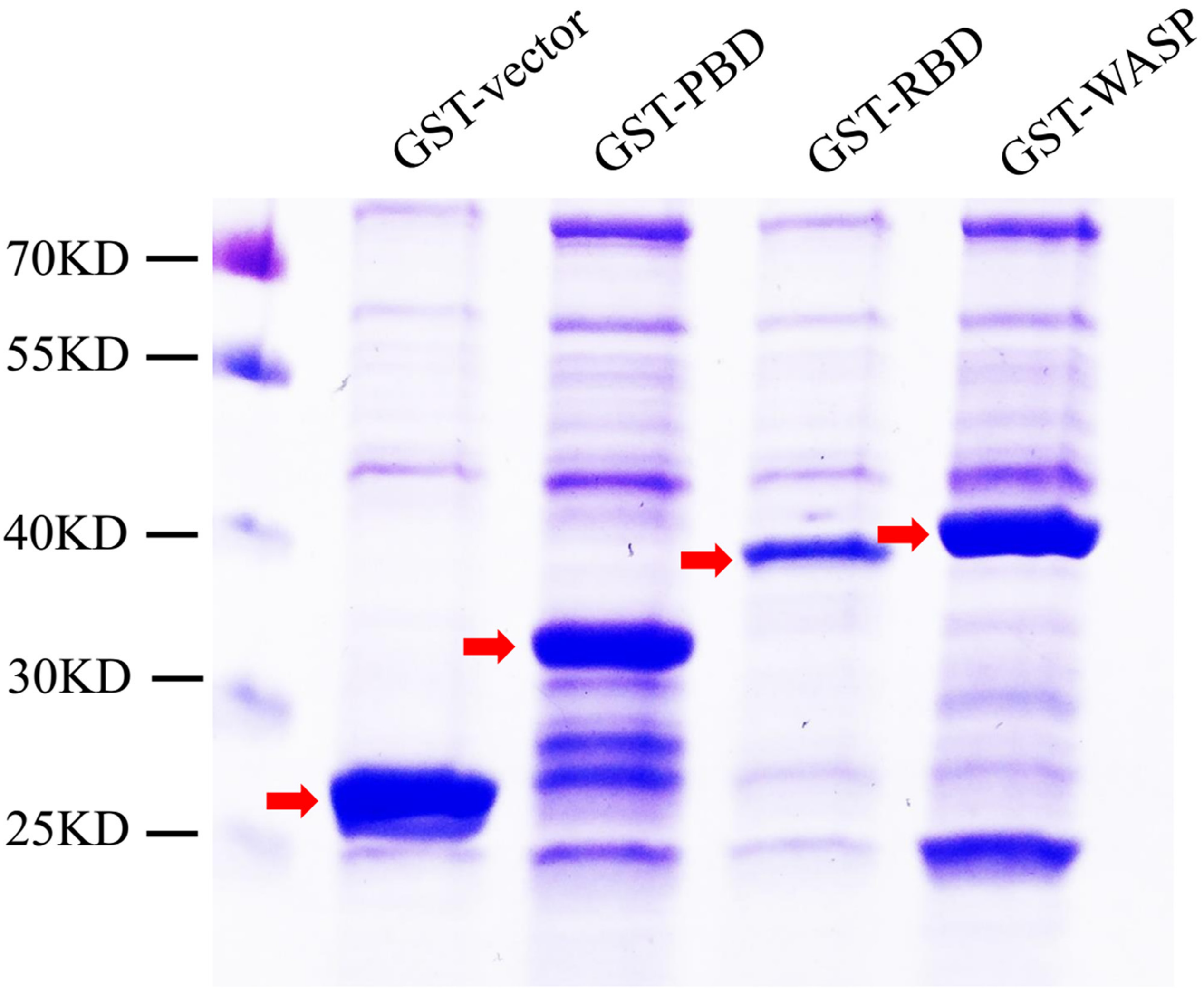

Supplementary Figure 3: The purified GST fusion protein of GST vector, PBD, RBD and WASP. The molecular weight of each protein were about 26kDa, 33kDa, 38kDa and 40kDa, respectively. Red arrow indicated the corresponding protein.

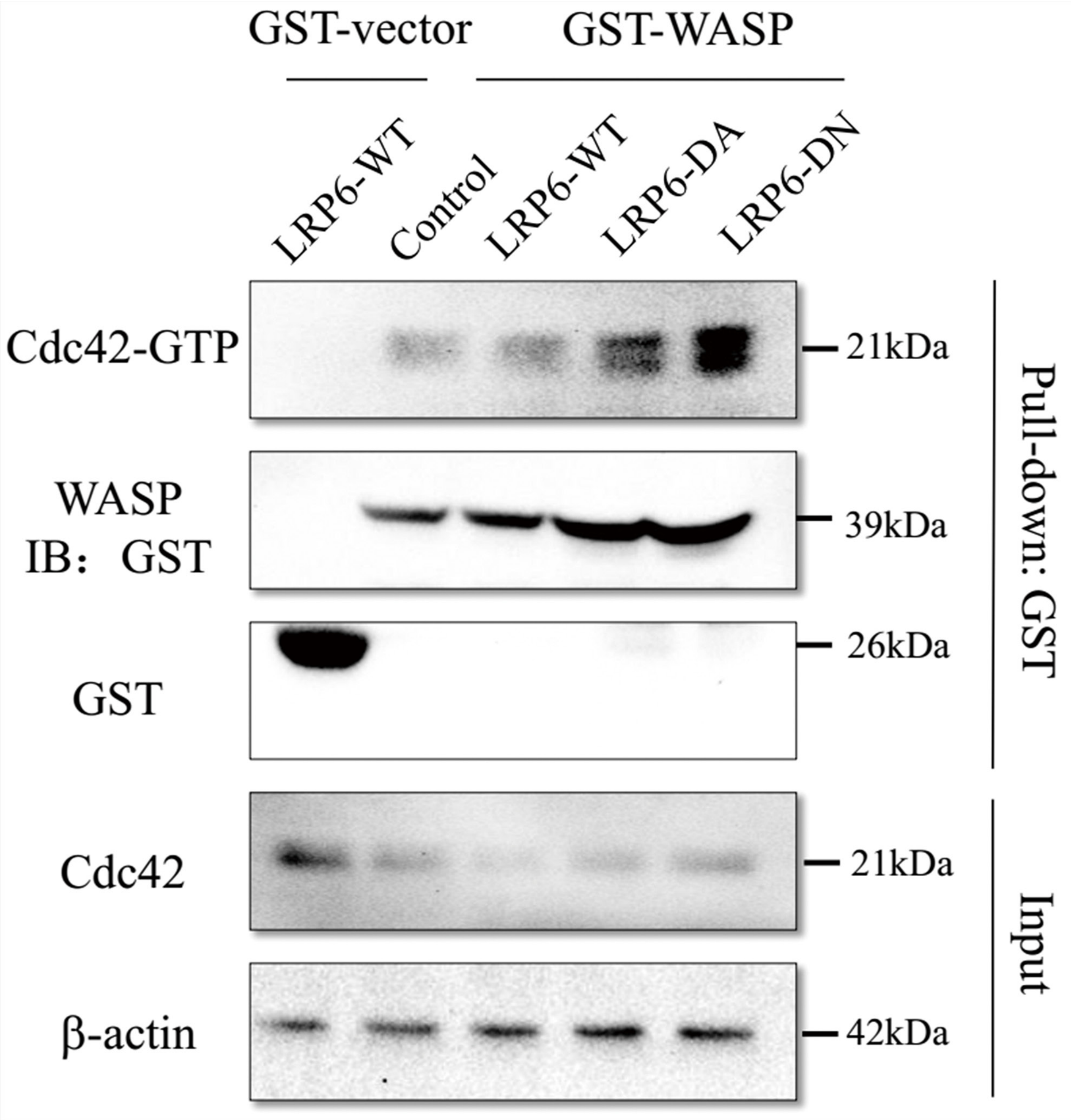

Supplementary Figure 4: The active GTP-bound Cdc42 level upon LRP6 overexpression. The GST fusion protein of WASP were expressed in BL21 cells and purified with glutathione agaroses. HCT116 cells were transfected with control vector, LRP6-WT, LRP6-DA and LRP6-DN and cell extracts were mixed with the produced agaroses. Then, the level of Cdc42 in GST pull-down lysate was evaluated by Western Blotting. The data showed was from HCT116.

Supplementary Table 1: Correlations between p-LRP6 immuno-staining and nuclear translocation of β-catenin in colorectal carcinoma

	p-LRP6 expression		Total	Significance	
	-	+		χ^2	*p* value
β-catenin nuclear translocation			183	6.458	0.011*
-	25	22			
+	44	92			

*p <0.05.

Supplementary Table 2: Correlations between p-LRP6 immuno-staining and clinicopathologic parameters in colorectal carcinoma

	Total	p-LRP6 expression		significance	
		Negative (%)	Positive (%)	χ^2	*p* value
Age				2.161	0.142
<60	64	28(43.8)	36(56.2)		
≥60	119	39(32.8)	80(67.2)		
Gender				0.036	0.850
Male	100	31(31)	52(52)		
Female	83	31(37.3)	52(62.7)		
Tumor size (cm)	150	4.06±1.878	4.64±2.107		0.657
Depth of invasion				36.801	<0.001***
≦T2	68	44(64.7)	24(35.3)		
T3-T4	115	23 (20)	92(80)		
Lymph node metastasis				12.995	<0.001***
No	80	41(51.3)	39(48.8)		
Yes	77	18(23.4)	59(76.6)		
Dukes stage				13.904	<0.001***
A-B	97	47(48.5)	50(51.5)		
C-D	85	19(22.4)	66(77.6)		
TNM stage				13.100	<0.001***
0-II	99	48(48.5)	51(51.5)		
III-IV	84	19(22.6)	65(77.4)		

***p<0.001.
